# Characteristics of Patients with Colonic Polyps Requiring Segmental Resection

**DOI:** 10.1155/2018/7046385

**Published:** 2018-02-11

**Authors:** Robert A. Mitchell, Chaoran Zhang, Cherry Galorport, Blair Walker, Jennifer Telford, Robert Enns

**Affiliations:** ^1^Department of Medicine, Division of Gastroenterology, University of British Columbia, Vancouver, BC, Canada; ^2^Department of Pathology and Laboratory Medicine, University of British Columbia, Vancouver, BC, Canada

## Abstract

**Background:**

It is unclear if the availability of new techniques for removal of large colonic polyps has affected the use of segmental colon resection. We sought to evaluate the characteristics of polyps undergoing surgical resection, including involvement of therapeutic gastroenterologists (TG).

**Methods:**

484 patients had a colonic resection; 165 (34%) were identified from the pathology database with polyp, adenoma, or mass in the clinical history field; these charts were reviewed.

**Results:**

128 patients (mean age 68 yrs, 72% male) were included. The mean polyp size was 2.9 cm (0.4 cm–12.0 cm). Adenocarcinoma was diagnosed in 50 (39.1%). 97 (75.8%) patients had a polyp that was felt to be unresectable by EMR, and 31 (24.2%) underwent successful EMR followed by surgery for adenocarcinoma (*n* = 29). The indication for surgery in those with unresectable polyps was variable and was not clearly documented in 51 (52.6%); only 17 of these patients (17.5%) had a TG involved.

**Conclusion:**

A high proportion of polyps managed by segmental resection did not contain adenocarcinoma. This data suggests that even in a tertiary care center where advanced endoscopic techniques are easily available, they are not always utilized. Educational endeavors to ensure that ideal pathways of intervention are utilized require implementation.

## 1. Introduction

Colorectal cancer (CRC) is the second most commonly diagnosed cancer in Canada, the second leading cause of death from cancer in men, and the third leading cause of death from cancer in women in Canada [1]. CRC has an estimated lifetime cost of approximately CAD$36,530 per patient [1]. Population-based screening programs in Canada recommend that patients with average CRC risk undergo annual or biannual testing with fecal immunochemical testing or fecal occult blood testing and patients who test positive go on to colonoscopy [2, 3]. Studies from Western nations show that screening reduces colorectal cancer mortality by up to 53% [4–6].

Endoscopic polypectomy at the time of colonoscopy is an effective means to prevent the development of CRC and death from CRC [7]. The majority of polyps (up to 90%) detected during colonoscopy are less than 10 mm and can be removed without significant operator difficulty using cold snare polypectomy [7]. Large polyps (usually defined as >10 mm) are much more challenging to remove endoscopically and usually require hot snare polypectomy with larger lesions often referred for surgical resection in Western nations. Large colonic polyps detected on colonoscopy are up to 5.5 times more likely than small polyps to undergo eventual surgical colectomy [8].

The standard of care for large colonic polyps in Eastern nations (namely Japan, China, and Korea) has been endoscopic mucosal resection (EMR) or, more recently, endoscopic submucosal dissection (ESD). EMR involves the expansion of submucosal space to create a plane for safe en bloc or piecemeal resection of colorectal polyps without injuring muscle [9], while ESD involves a larger en bloc resection which often includes submucosal tissue [10]. EMR has been accepted as an effective treatment for large colorectal polyps [11–13]. Although no Canadian guideline recommends EMR as an intervention for large colonic polyps, the recent European Society of Gastrointestinal Endoscopy (ESGE) recommends EMR for nonpedunculated, noninvasive colonic polyps over 10 mm [14].

At present, a clear knowledge gap exists with respect to patients in Canada that are found to have large colonic polyps. There are no consensus guidelines in Canada yet on how to refer and treat these patients. ESD is not yet a widely used technique in most Canadian centers and it is suspected that many large polyps are referred to surgical resection with minimal therapeutic gastroenterologist involvement, or attempts at EMR. It is unclear how the development of techniques such as EMR has affected referral patterns and segmental colon resection for large colonic polyps. As more patients are referred for colonoscopy from provincial CRC screening programs, it is important to know which patients are being referred for surgical resection and whether advanced endoscopic techniques for polypectomy have been attempted prior to surgical resection to ensure that the latest techniques for management of these polyps are being employed to maximize the benefit to the patient.

## 2. Methods

### 2.1. Patient Selection

**Figure 1:**
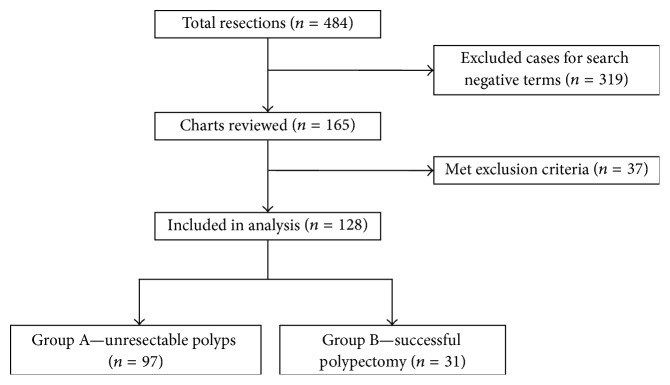
Patients included and excluded from a retrospective chart review of patients undergoing segmental colonic resection at St. Paul's Hospital in Vancouver, Canada. Inclusion and exclusion of patients in a retrospective chart review of characteristics of patients who have undergone segmental colonic resection for polyps.

This retrospective cohort study was conducted at St. Paul's Hospital in Vancouver, British Columbia. St. Paul's Hospital is a tertiary referral center in downtown Vancouver. All patients in this study were selected from a pathology database of patients who had colonic resection samples submitted to pathology from January 1, 2010, to December 31, 2014. The total number of individual patients in this database was 484. In these 484 patients, search terms (“polyp”; “adenoma”; “mass”) were applied in the clinical history field of pathology requests to identify patients whose indication for surgery was a polyp, adenoma, or mass within the colon. 165 patients (34%) were identified from the pathology database with “polyp”, “adenoma”, or “mass” in the clinical history field; these 165 charts were reviewed in full. Exclusion criteria were obstructing colon mass noted on colonoscopy report; polyposis syndrome; prior colorectal cancer; postpolypectomy perforation; and transanal endoscopic microsurgery. 37 cases met exclusion criteria (Figure 1). This study was approved by an institutional review board at the University of British Columbia and St. Paul's Hospital.

### 2.2. Data Collection

Clinical data was obtained from patients' operative notes, clinical notes, and referral letters. Pathological data was obtained from pathology reports for each segmental colonic resection and individual polyp pathology submissions. Data regarding details of endoscopic procedures was obtained from endoscopic reports. “Therapeutic gastroenterologist” for the purposes of this study was defined as a gastroenterologist who has completed an advanced endoscopy fellowship. A presurgical diagnosis of adenocarcinoma in this study was based on pathology from endoscopic biopsy or polyp resection specimens.

### 2.3. Statistical Analysis

Continuous variables were reported as median (range) and discrete variables were expressed as *n* (%) unless otherwise specified. Data analysis was conducted using statistical software (SPSS Statistics® v22, IBM, Armonk, NY, USA). Fisher's exact test was used to compare categorical variables. The Student's *t*-test was used to compare continuous variables. *p* values were calculated as 2-tailed and a value of ≤0.05 was interpreted as significant.

## 3. Results

### 3.1. Patient Characteristics

One-hundred and twenty-eight individual patients were included in the final study analysis. The mean patient age was 67.8 years (37–87 years). Most study subjects were male (*n* = 93, 71.9%). Nineteen patients reported a first-degree relative with a history of colorectal cancer (14.8%). Three patients reported a diagnosis of inflammatory bowel disease (2.3%) (Table 1).

### 3.2. General Polyp and Surgical Characteristics

Fifty-five patients had polyps that were noted to be sessile on endoscopy report (39.8%). The mean polyp size for patients included in this study was 28.9 mm (±19.3 mm). The location of the polyp was most commonly in the cecum/ascending colon (*n* = 89, 69.5%). Ninety-seven patients had polyps that were deemed to be unresectable on initial endoscopy (Group A—75.8%) and 31 patients had polyps that were successfully resected by colonoscopy (Group B—24.2%). The most common operation performed was right hemicolectomy (*n* = 85, 66.4%), followed by rectosigmoid resection (*n* = 19, 14.8%), left hemicolectomy (*n* = 9, 7.0%), total colectomy (*n* = 3, 2.3%), and other segmental resections (*n* = 12, 9.4%). A final diagnosis of adenocarcinoma was made in 50 patients (39.1%). This includes patients with adenocarcinoma diagnosed only on polyp biopsy/attempted EMR or resection (*n* = 20, 15.6%) or only in final surgical specimens (*n* = 11, 8.6%) or contained within both resected/biopsied polyps and final surgical specimens (*n* = 19, 14.8%).

### 3.3. Characteristics of Unresectable Polyps (Group A)

Of the 97 patients with polyps deemed to be unresectable who went on to eventual colonic resection, a specific reason for failure of endoscopic polyp resection was not clearly documented in 51 (52.6%). Among those patients with documented reasons for unresectable polyps, the most common reason for endoscopic failure and subsequent surgical resection was polyp size or location (*n* = 31, 32.0%), one attempted polypectomy which was abandoned and not reattempted (11, 11.3%), concurrent diverticulosis (2, 2.1%), or poor polyp visualization due to patient factors (2, 2.0%). Within this group, 38 polyps were sessile (39.2%) and 21 contained adenocarcinoma (21.6%) on final surgical pathology. Of the 97 patients with polyps deemed endoscopically unresectable, only 17 (17.5%) had a therapeutic gastroenterologist involved in their care. Within this group, adenocarcinoma was detected prior to surgery in 10 patients (10.3%) by partial polypectomy or polyp biopsy. In this group, a diagnosis of adenocarcinoma was made in 11 patients after surgery by examination of final surgical specimen (11.3%).

### 3.4. Characteristics of Resectable Polyps (Group B)

Of the 31 patients with resectable polyps who went on to surgical colonic resection, the most common reason for surgical resection was adenocarcinoma on polyp pathology in 29 (93.6%). Far less common reasons for surgery in this group included technical difficulty with colonoscopy and concerns for surveillance (1 patient, 3.2%), and concomitant colitis requiring resection (1 patient, 3.2%). Of the 31 patients in this group, 9 had a therapeutic gastroenterologist involved in their care (29.0%). Within this group, all 29 patients had adenocarcinoma detected by polypectomy prior to surgery. Of these 29 patients, 11 also had residual adenocarcinoma detected within their final surgical resection specimen (37.9%).

### 3.5. Comparison between Unresectable and Resectable Polyps

Polyps in Group A had a larger mean diameter than Group B, (31.8 ± 19.9 mm versus 18.2 ± 12.4 mm, *p* < 0.01). Successful polyp resection prior to surgery was not affected by polyp morphology (41.9% sessile resectable, 39.2% sessile unresectable, *p* = 0.83). A final diagnosis of colorectal adenocarcinoma was made in 50 patients (39.1%). Adenocarcinoma was present in 21 patients in Group A (21.6%) and 29 patients in Group B (93.5%) (*p* < 0.01) (Table 2). A presurgical diagnosis of adenocarcinoma was made in 10 patients in Group A (10.3%), and 29 patients in Group B (93.5%) (*p* < 0.01). A new postsurgical diagnosis of adenocarcinoma was made in 11 Group A patients (11.3%) and no Group B patients (0%) (*p* = 0.06).

## 4. Discussion

Endoscopic polypectomy at the time of colonoscopy is an effective means to prevent the development of CRC and death from CRC [8]. Large colonic polyps encountered on colonoscopy have classically been associated with a higher risk of colonic resection than small polyps [9]. Well-validated endoscopic methods, such as EMR, have been developed to safely perform polypectomy on larger colonic polyps [11, 15, 16]. It is unclear if the advent of these methods has affected the use of segmental resection or colectomy for colonic polyps. We, therefore, sought to evaluate the characteristics of polyps undergoing surgical resection, including the involvement of therapeutic gastroenterologists. Our results show that a high proportion of patients undergo colonic resection for polyps that are deemed unresectable by endoscopic methods, and that many polyps managed by surgical resection do not contain adenocarcinoma. Furthermore, we demonstrate that a low proportion of patients with polyps managed by surgical resection have a therapeutic gastroenterologist in their cascade of care prior to undergoing surgery.

To our knowledge this is the first published study reporting attempts, referral patterns, and reasons for failure of polypectomy in patients undergoing surgical resection for large colonic polyps. The findings of a high rate of cancer in these polyps is comforting; however, the fact that many of these polyps were not assessed by experts comfortable with EMR is concerning and clearly suggests that rapid access to surgery was the pathway most commonly selected. Several large studies and subsequent systematic reviews have demonstrated the safety and effectiveness of polypectomy as an alternative to surgical resection of large polyps [16–18]. In a large multicenter trial by Moss et al., polypectomy of large polyps was attempted with complete excision achieved in 89.2% of cases (414/464) [16]. The study reported that, of the 50 failed cases, 25 (50%) went on to receive eventual segmental resection. The rate of successful polypectomy in our study was much lower at 24.2%. This is surprising, but is in part due to our study methodology—identifying patients from a population who required segmental resection for colonic polyps and retrospectively examining success of polypectomy. Among the 75.8% patients in our sample who required surgery because of unresectable polyps, a clear reason for why polyps were unresectable was not documented in 52.6%. It is possible that in some of these cases there was an initial concern for deep submucosal invasion that caused the endoscopist not to attempt polypectomy and refer the patient directly for surgical resection. The Paris endoscopic polyp classification and Kudo pit surface pattern classification systems allow for morphological classification of polyps at the time of endoscopy and may be useful in the prediction of deep submucosal invasion [16, 19]. At the time that data was acquired for this study, however, polyp morphology was not reported on most endoscopic reports in British Columbia. This has since been incorporated as a standard part of endoscopic reporting of polyps in British Columbia and will undoubtedly play a role in the management of large polyps going forward.

There may also have been undocumented concerns related to size and location of the polyp. In the report by Moss et al., 40.9% of patients had a lesion that was deemed to be in a position that was difficult to reach or difficult to resect, or both [16]. In our study, this was documented in only 32.0% of patients. It is possible that some of the patients in our study who did not have documented reasons for unresectable polyps had polyps that were in a location or of a size that made resection challenging, and that this was simply not noted on the colonoscopy report.

Only a small number of polyps deemed unresectable at colonoscopy had polypectomy attempted by therapeutic gastroenterologist trained in advanced EMR. This data suggests that even in a tertiary care center with advanced endoscopic techniques easily available, they are not always utilized. The reasons for this are not clear at present. As noted above, we believe that it is likely that initial endoscopists harbored concerns around submucosal invasion of larger polyps resected by EMR and therefore tended to refer directly to surgical resection rather than a therapeutic gastroenterologist for EMR. EMR for large polyps is usually performed in a piecemeal fashion, limiting adequate pathological examination, which in turn may lead to an inability to identify sites of focal submucosal invasion with subsequent local recurrence or an underestimation of metastatic risk [20]. It appears that piecemeal resection may be a considerable risk factor for local recurrence compared to en bloc resection [21, 22] and may underlie some colonoscopists' reluctance to perform piecemeal EMR for larger polyps. Further study, perhaps in the form of a survey, would be required to determine the specific reasons for colonoscopists not involving therapeutic gastroenterologists with expertise in EMR, over direct referral for surgical resection.

A high proportion of polyps managed by segmental resection in our study did not contain adenocarcinoma. Over half of the patients in Group A with eventual adenocarcinoma diagnosed had the diagnosis made after surgical resection in the final biopsy specimen. Surgery was likely performed in Group B patients due to detection of cancer on pathology and concern for deep invasion. As outlined above, although many of these patients had large polyps successfully removed, a piecemeal resection technique limited adequate histological examination of the resected polyp specimen to exclude submucosal invasion. In the future, more therapeutic gastroenterologists in Canada may gain experience with ESD which allows for en bloc resection of larger polyps and full histologic examination to exclude submucosal invasion. Using such a technique in Group B patients from this study would allow for confidence of complete resection with no residual adenocarcinoma, and therefore no need for further surgery. Recent guidelines from the Endoscopic Forum Japan 2016 recommend EMR for polyps measuring 10 mm to 20 mm and ESD for polyps greater than 20 mm [17]. Although ESD is shown to have greater chance of en bloc resection compared to EMR, it is associated with a higher risk of perforation [23]. As more Canadian endoscopists gain experience with ESD, these risks and benefits will have to be considered in the context of individual patients, but conceivably such a technique would negate the need for surgical resection in some of the 18 of 29 Group B patients who did not have residual adenocarcinoma detected in their final surgical specimen. Additionally, patients with invasion of adenocarcinoma into the superficial submucosa (sm1) may be candidates for ESD, which may further reduce the need for surgical resection even in those patients with a diagnosed adenocarcinoma [14].

Interestingly, the majority of segmental colonic resections in this study were performed for right colonic polyps (69.5%). This is more than those in the study by Moss et al., where 45.9% had polyps successfully removed from the right side of the colon [16]. The high rate of segmental resection for right-sided polyps in our sample may be due to the relative difficulty of polypectomy in the right colon compared to the left leading to more failed polypectomies and subsequent surgeries. Conversely there were few rectal cases, perhaps due to relative ease of polypectomy in the rectum and exclusion of transanal micro cases which often deals with rectal lesions and to which access is easily present at this institution.

Our study has several limitations. First, it is retrospective in design. Second, this study includes review of pathological data from a single tertiary care center. It would be fascinating to measure differences in referral patterns for colonic resection of polyps in community or rural health care centers where access to therapeutic gastroenterologists may be even more limited. Third, the impact of quality of bowel preparation on rates of surgical referral or success of polyp resection was not analyzed in this study. At the time when data was acquired for this study, this parameter was not recorded as part of standard endoscopic reporting in our center. Since then, this has been suggested as part of standard endoscopic reporting in British Columbia and is currently included in endoscopic reports at our center. Finally, this study includes data drawn over a relatively long period of time. The popularity and comfort level of endoscopic techniques like EMR may have evolved over the four-year study period within our region, and there may have been increasing rates of successful polypectomy or referral to therapeutic gastroenterologists over the study time frame.

Nevertheless, the present study captures important real-world data on patients who require segmental resection for polyps and provides valuable information on the need to educate physicians on the role of therapeutic gastroenterologists in the management of larger polyps with EMR presently, and ESD in the future. This study also highlights the need for clearer and more specific endoscopic documentation regarding reasons for polypectomy failure. With the advent of advanced endoscopic techniques to remove large polyps and the expertise developing throughout Canada for the management of these cases, educational maneuvers to ensure ideal patient care are required.

## Figures and Tables

**Table 1:** Patient characteristics at segmental resection performed for large colonic polyps at St. Paul's Hospital in Vancouver, Canada.

	Patients (*N* = 128)	
	*n*	(%)
Gender, male	93	(71.9%)
Age, years (range)	67.8 (37–87)	
Family history of colorectal cancer	19	(14.8%)
History of IBD	3	(2.3%)
Sessile polyps	55	(39.8%)
Polyp size, mm ± SD	28.9 ± 19.3	
Location of polyp	-	
Ascending/cecum	89	(69.5%)
Transverse	4	(3.1%)
Descending	5	(3.9%)
Sigmoid	17	(13.3%)
Rectal	5	(3.9%)
Unclear or more than one concerning polyp	8	(6.3%)
Operation performed	-	
Right hemicolectomy	85	(66.4%)
Left hemicolectomy	9	(7.0%)
Rectosigmoid resection	19	(14.8%)
Total colectomy	3	(2.3%)
Segmental resection (other)	12	(9.4%)

Characteristics of patients at the time of segmental resection for large colonic polyps. IBD: inflammatory bowel disease; SD: standard deviation.

**Table 2:** Comparison of resectable and unresectable polyps in patients undergoing eventual colonic segmental resection at St. Paul's Hospital in Vancouver, Canada.

	Group A unresectable (*n* = 97)	Group B resectable (*n* = 31)	*p* value
Mean polyp size, mm ± SD	31.8 ± 19.9	18.2 ± 12.4	<0.01
Polyp pathology, sessile	38 (39.2%)	13 (41.9%)	0.83
Adenocarcinoma	21 (21.6%)	29 (93.5%)	<0.01

Comparison of polyps that were deemed resectable or unresectable in patients who eventually had segmental colonic resection. SD: standard deviation.
